# The nuclear envelope: target and mediator of the apoptotic process

**DOI:** 10.1038/s41420-020-0256-5

**Published:** 2020-04-27

**Authors:** Liora Lindenboim, Hila Zohar, Howard J. Worman, Reuven Stein

**Affiliations:** 1 Department of Neurobiology, School of Neurobiology, Biochemistry and Biophysics, George S. Wise Faculty of Life Sciences, Tel Aviv University, 69978 Ramat Aviv, Israel; 2 Department of Medicine and Department of Pathology and Cell Biology, Vagelos College of Physicians and Surgeons, Columbia University, New York, NY 10032 USA

**Keywords:** Apoptosis, Proteases

## Abstract

Apoptosis is characterized by the destruction of essential cell organelles, including the cell nucleus. The nuclear envelope (NE) separates the nuclear interior from the cytosol. During apoptosis, the apoptotic machinery, in particular caspases, increases NE permeability by cleaving its proteins, such as those of the nuclear pore complex (NPC) and the nuclear lamina. This in turns leads to passive diffusion of cytosolic apoptogenic proteins, such as caspases and nucleases, through NPCs into the nucleus and the subsequent breakdown of the NE and destruction of the nucleus. However, NE leakiness at early stages of the apoptotic process can also occur in a caspase-independent manner, where Bax, by a non-canonical action, promotes transient and repetitive localized generation and subsequent rupture of nuclear protein-filled nuclear bubbles. This NE rupture leads to discharge of apoptogenic nuclear proteins from the nucleus to the cytosol, a process that can contribute to the death process. Therefore, the NE may play a role as mediator of cell death at early stages of apoptosis. The NE can also serve as a platform for assembly of complexes that regulate the death process. Thus, the NE should be viewed as both a mediator of the cell death process and a target.

## Facts

- The NE is an important target of the apoptotic machinery.
- Caspase-dependent targeting of the nuclear envelope increases nuclear envelope permeability to cytosolic apoptogenic factors, which then promote the apoptotic manifestation of nuclear destruction.
- The apoptotic process can also perturb the integrity of the nuclear envelope by a caspase-independent, but Bax-dependent process, which causes transient and repetitive local generation and rupture of nuclear protein-containing nuclear bubbles (GRUNB). The rupture of these bubbles leads to redistribution of the nuclear proteins to the cytosol that might subsequently act as amplifiers of the apoptotic process.
- The NE can also regulate the cell death process by anti-or pro-apoptotic activity of some nuclear proteins as well as by severing as a platform for assembly of apoptotic complexes.

## Open questions

- How does activated Bax cause generation and rupture of nuclear bubbles?
- Which nuclear proteins are redistributed during stress-induced GRUNB (SIGRUNB) and what is their relative contribution to the apoptotic process?
- Can Bax be involved in GRUNB under non-apoptotic conditions?
- Does the Linker of Nucleoskeleton and Cytoskeleton (LINC) complex play a role in SIGRUNB?
- Can SIGRUNB promote non-apoptotic cell death?
- Does SIGRUNB have a physiological role?

## Introduction

**Fig. 1:**
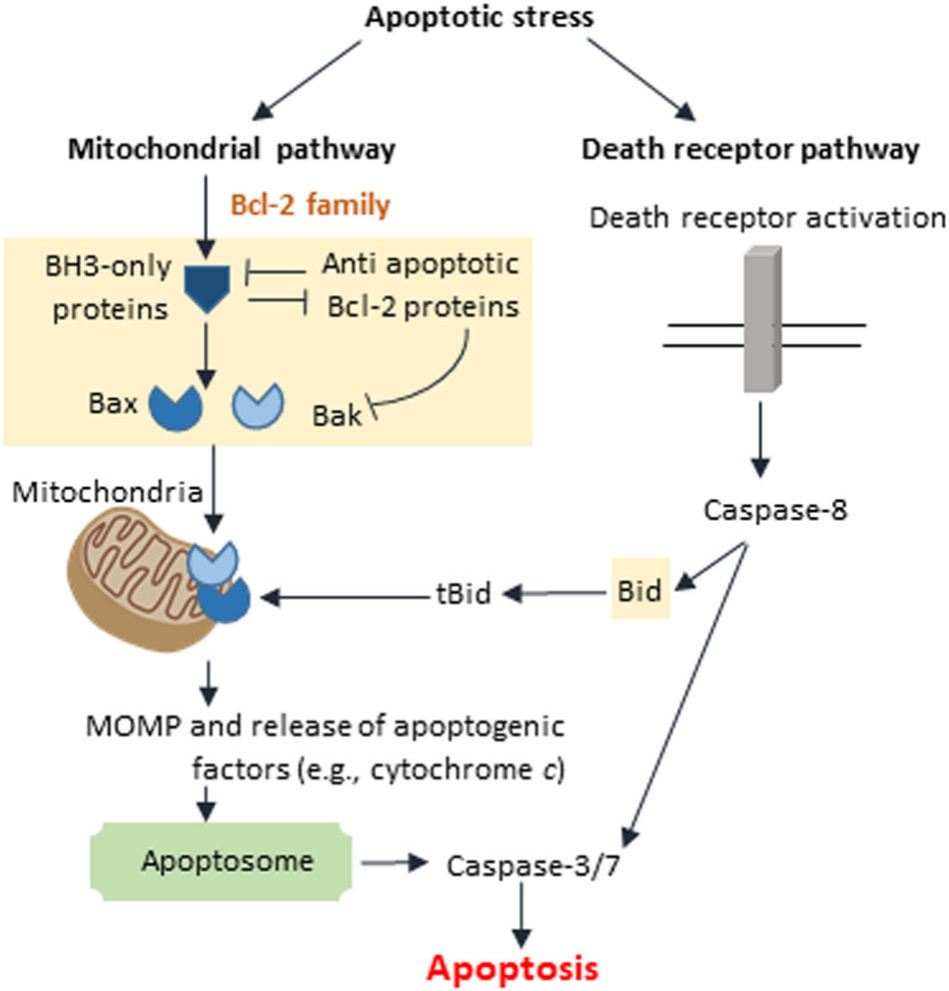
Schematic representation of the apoptotic pathways. Apoptosis is executed in cells by two main pathways, the mitochondrial (intrinsic) and death receptor (extrinsic) pathways. The mitochondrial pathway is regulated by the Bcl-2 family proteins. Under apoptotic stress, the pro-apoptotic BH3-only proteins act by inhibiting anti-apoptotic Bcl-2 proteins, thereby releasing the effector pro-apoptotic Bax and Bak proteins from anti-apoptotic protein inhibition, and/or by directly activating Bax and Bak. Activated Bax and Bak then undergo conformational changes, which leads to their oligomerization and the subsequent mitochondrial outer membrane permeabilization (MOMP), followed by the release of apoptogenic factors such as cytochrome *c* to the cytosol. Cytochrome *c* then binds to Aapf1 and promotes generation of the caspase-activating platform apoptosome. This leads to caspas-9 activation and the subsequent activation of the executer caspase-3/7, leading to apoptotic death. In the death receptor pathway, following ligand binding to cell membrane death receptors, the death-inducing signaling complex (DISC) is formed and caspase-8 is activated. Activated caspas-8 can promote cell death by directly activating caspase-3/7 or cleaving the pro-apoptotic BH3-only protein Bid. The product of this cleavage, tBid, then converges to the mitochondrial pathway by activating Bax/Bak.

The apoptotic process is characterized by a series of sequential events that culminate in cell death. Two main protein families regulate and execute the cell death process: the Bcl-2 proteins family and caspases (Fig. 1). The Bcl-2 proteins family acts mainly, but not only, by regulating the integrity of mitochondria. It contains pro-apoptotic and pro-survival proteins. The pro-apoptotic sub-family is comprised of the multi-domain proteins Bax and Bak as well as the Bcl-2 homology domain 3-only (BH3)-only proteins such as Bid and Bim. The pro-survival proteins such as Bcl-2 and Bcl-x_L_ bind to and block the activity of the pro-apoptotic proteins. Bax and Bak promote mitochondrial outer membrane permeabilization (MOMP) and release of apoptogenic factors from the mitochondrial inter-membrane space. This leads to caspase activation via the apoptosome and by inhibiting the anti-caspase activity of inhibitor of apoptosis protein family members. The BH-3 only proteins and the pro-survival proteins regulate Bax/Bak activity by promoting or inhibiting Bax/Bak activation, respectively (for review see refs. ^1,2^ and references therein). In addition to the mitochondria, the different Bcl-2 family members can reside in various subcellular localizations including endoplasmic reticulum (ER) (e.g., Bcl-2, Bcl-x_L_, Bok, and Bax), the nucleus (e.g., Bid, and Bcl-2), and the NE (e.g., Bcl-2, Bcl-x_L_)^1,3^.

Caspases are cysteine aspartate proteases that cleave a subset of essential cellular proteins to promote apoptotic cell death. They are categorized into two groups, initiator caspases (caspase-2, caspase-8, and caspase-9) and effector caspase (caspase-3, caspase-6, and caspase-7). In response to apoptotic stress, trigger-specific complexes are assembled, to which distinct initiator caspases are recruited and activated. The activated initiator caspases cleave and thereby activate the effector caspases. The effector caspases then execute the death process (for review see refs. ^4,5^ and references therein). Activation of the death receptor pathway leads to activation of caspase-8. In some cells, caspase-8 can cleave the BH3-protein Bid to generate truncated Bid (tBid), which then induces the mitochondrial pathway, linking the death receptor pathway to the mitochondrial pathway. Caspases can also promote non-apoptotic effects such as inflammation (For review see refs. ^6–8^ and referenced therein).

The apoptotic process demolish the cell via multiple pathways that lead inter alia to destruction of essential cell oranges such as the Golgi, mitochondria, and the nucleus. The nuclear destruction is one of the hallmarks of apoptosis and it consists of degradation of nuclear DNA, chromatin condensation, nuclear fragmentation, and NE collapse^9,10^. The NE is a central target of the apoptotic machinery and apoptosis-induced alteration in NE is an important early event in the demolition of the nucleus^11–13^.

**Fig. 2:**
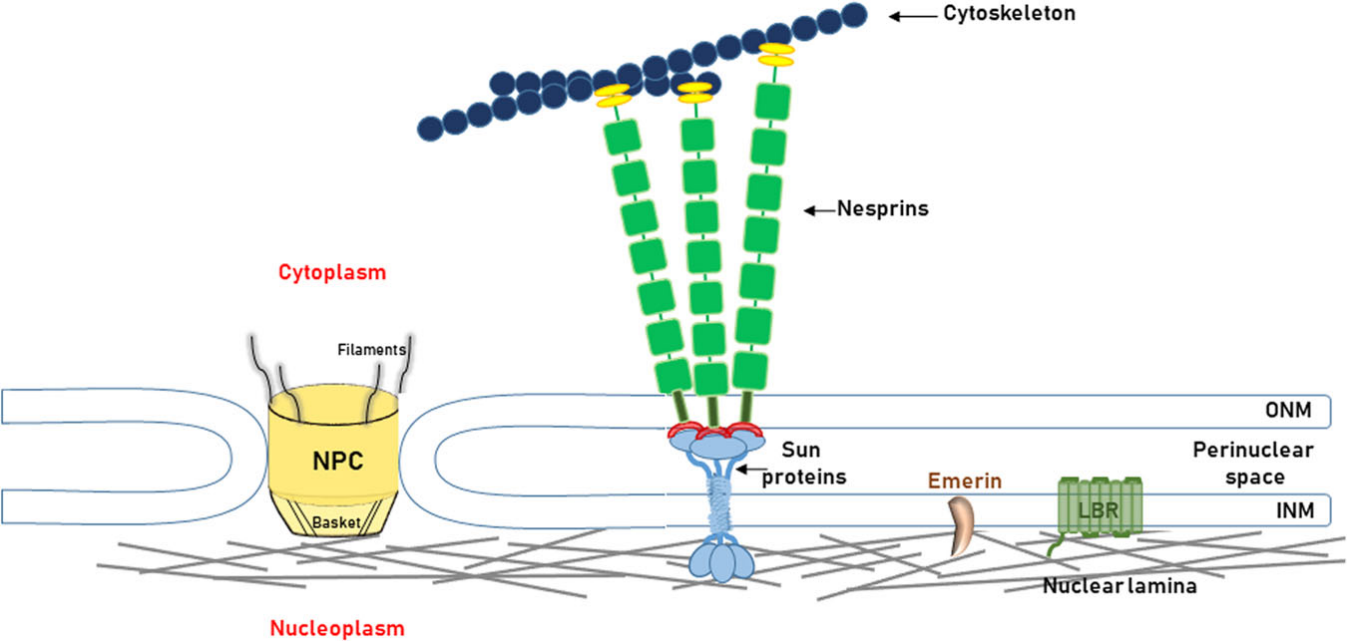
Schematic representation of the nuclear envelope. The nuclear envelope is composed of the outer (ONM) and inner (INM) nuclear membranes, the nuclear lamina and the NPCs. NPCs span both membranes and allow for passive and active transport of materials into and out of the nucleus. NPCs contain nuclear baskets that project into the nucleoplasm and filaments that project into the cytoplasm. Like many integral proteins of the inner nuclear membrane, lamin B receptor (LBR) and emerin (shown as examples) bind to the nuclear lamina and/or chromatin. SUN proteins, other integral proteins of the INM, have N′ termini localized in the nucleoplasm, where they bind to the nuclear lamina, and C′ termini containing SUN domains localized in the perinuclear space. Nesprin proteins contain spectrin repeats (light green), calponin-homology domains (yellow), which interact with actin fibers, a transmembrane domain (dark green), which spans the ONM, and a KASH domain (red), which interacts with sun domains of SUN proteins. SUN and nesprin proteins form the core of the LINC complex.

The NE (Fig. 2) defines the nuclear boundaries in eukaryotic cells and provides the nucleus with architectural and mechanical support. It consists of the nuclear membranes, nuclear lamina, and NPCs. The outer nuclear membrane (ONM), which is continuous with the ER membrane, and the inner nuclear membrane (INM) are separated by the perinuclear space, a continuation of the ER lumen. NPCs are located at sites where the ONM and INM merge and are the passageways for passive and active transport across the NE^14^. The INM is underlined by the nuclear lamina, a meshwork of type V intermediate filament proteins called lamins^15^. The nuclear lamina interacts with the chromatin and with integral INM proteins such as lamin B receptor (LBR) and emerin, as well as several others. In addition, the lamina serves as a chromatin organizer and participates in signal transduction between the cytoskeleton and the nucleus^16,17^. The LINC complex connects the nuclear interior with the cytoskeleton^18^. It is composed of Klarsicht/ANC-1/Syne-1 homology (KASH) domain proteins, called nesprins in mammals, and Sad1p, UNC-84 (SUN) domain proteins called SUNs. Nesprins are transmembrane proteins of the ONM that bind directly or indirectly to actin, microtubules, or intermediate filaments in the cytoplasm. SUNs are transmembrane proteins of the INM that bind to lamins inside the nucleus. The KASH domain of nesprins is in the perinuclear space, where it interacts with the SUN domain of SUN proteins, forming a bridge between the nuclear lamina and the cytoskeleton^19,20^.

The NE is affected by and affects the apoptotic process in multiple ways. These include caspase-dependent and -independent alterations of NPCs^11^ as well as caspase-dependent cleavage of lamins and other NE proteins. These processes increase NE permeability to cytosolic apoptotic factors such as caspases and nucleases. These enter the nucleus, destroying its integrity and leading to NE breakdown, chromatin condensation, and DNA fragmentation. In response to apoptotic stress, the NE can also undergo transient and repetitive ruptures that involve Bax-dependent but caspase-independent generation and subsequent rupture of nuclear bubbles (GRUNB)^21^. This stress-induced GRUNB (SIGRUNB) leads to redistribution of nuclear proteins from the nucleus to the cytosol where some of them may participate in the apoptotic process. In addition, the NE can serve as a platform for the assembly of apoptotic complexes and NE proteins can have pro- and anti-apoptotic activities. Therefore, the NE can have dual roles in apoptosis, namely, as a target and a mediator.

The aim of this review is to provide an overview of the role of the NE in apoptosis, emphasizing that it is not only a target for breakdown in the apoptotic process, but also has the potential to function as a mediator and a promoting agent of the process. Previous reviews on the NE during apoptosis have focused mainly on it being a target of the apoptotic machinery, particularly on the effect of caspases on NPCs and the resulting effects on nucleocytoplasmic transport^11,22,23^. This review will focus primarily on caspase-independent effects on the NE during apoptosis, emphasizing SIGRUNB, and on the role of the NE as a mediator of the apoptotic process.

## The NE as a target of the apoptotic machinery

### Apoptosis-induced increase in NE permeability

The NE separates the nucleus from the cytoplasm and allows the passage of selected cargo in and out of the nucleus through NPCs. In response to apoptotic triggers, the NE undergoes changes including clustering of NPCs^24^, detachment of the nuclear membrane from chromatin^12^ and an increase in permeability, which is mediated by caspase-dependent and-independent alterations of the NPCs. This results in the passive diffusion of apoptotic proteins such as caspase-3^25^, apoptosis inducing factor^26^, and cytochrome *c*^27,28^ into the nucleus.

#### Caspase-dependent effects on the NE proteins

##### Cleavage of NPC proteins

Among the mechanisms governing NE leakiness during apoptosis is an increase in the permeability of NPCs induced by caspases^29^ such as caspas-2, caspase-3, and caspas-9 (see Shahin^22^ and references therein). The cleavage by caspases of a subset of proteins that compose the NPC – nucleoporins p62^30^, Pom121^31,32^, Nup93 and Nup96^33^, RanBP2/Nup358, Nup214 and Nup153^30,34^, and Tpr^34^ – leads to an alteration in NPC permeability. Notably, this cleavage is not arbitrary. It occurs in a minimalist but effective manner in which only distinct components of the NPC are degraded^33^. Accordingly, the NPC central core is not subjected to massive cleavage by caspases, where only the proteins Nup93 and Nup96 are cleaved. On the other hand, many peripheral nucleoporins, both at the cytoplasmic and nuclear sides of the NPC, are cleaved by caspases. This notion is supported by nano-imaging using atomic force microscopy (AFM), which has shown that in apoptotic *Xenopus laevis* oocytes, NPCs either partially or completely lose their nuclear basket and cytoplasmic filaments^35^. Nups can also be cleaved by calpains in addition to caspases^36^.

##### Cleavage of nuclear lamina and INM proteins

Besides the NPC, additional NE proteins are cleaved by caspases during apoptosis. These include the nuclear lamina proteins lamin A/C and B-type lamins^30,31^, which are cleaved by caspase-6^12^, as well as the lamin-associated proteins LAP2α and LAP2β^30,37^. LAP2α, LAP2β, and lamins function in attaching chromatin to the INM^15^. Therefore, their cleavages would be expected to detach the NE from chromatin, a process that occurs during nuclear destruction during apoptosis^12^. Caspases can also cleave LBR^38^ and emerin^39,40^ in some but not all^30,37^ apoptotic cells. Caspases may also affect nuclear lamina integrity indirectly by cleaving and activating protein kinase C (PKC)-δ. Activated PKC-δ then translocates to the nucleus where it binds and phosphorylates B-type lamins. This phosphorylation together with caspase-mediated cleavage of B-type lamins can dismantle the lamina^41^.

#### Caspase-dependent NE breakdown

Transmission electron microscopy (TEM) has demonstrated that the structural integrity of the NE is largely maintained during apoptosis^10,30^. In addition, assessment of NE leakiness in isolated *Xenopus laevis* oocytes nuclei has shown that the apoptosis-induced NE leakiness is prevented by blocking the NPC channel^42^. NE leakiness, at least in this in vitro model, is through the NPCs. However, NE leakiness can also occur as a result NE breakdown. NE breakdown was detected in apoptotic cells using TEM^43,44^ or carbon nanotube AFM probe systems^45^. The latter study showed that NE rupture was associated with DNA leakage out of the nucleus and that these effects occurred in a caspase-dependent manner^45^. The notion that NE breakdown during apoptosis is mediated by caspases is supported by an additional study^44^, which suggests that this effect is mediated by a caspase-dependent cleavage of C53/LZAP (CDK5RAP3), a protein that has been implicated in various signaling pathways. This study showed that ectopic expression of C53/LZAP-C3 peptide, which corresponds to the C-terminal fragment generated by caspase-dependent cleavage of C53/LZAP, promoted NE breakdown, DNA herniation, and nuclear irregularity. NE breakage that was associated with the appearance of spherical membrane buds devoid of chromatin and NPC clustering was also observed in apoptotic human T lymphoblastic KE37 cells^30^. These effects were associated with caspase-dependent cleavage of lamin B2, LAP2, and Nup153, suggesting that they contribute to NE breakage^30^.

#### Caspase-independent effect on the NE

Proteolysis of NPC components is not the only mechanism that causes NE permeabilization. Nuclear leakiness can occur early in the apoptotic cascade, before caspase activation or MOMP and without cleavage of NPC proteins. This early leakiness is associated with influx of large molecules (4xCherry/104 kDa) into the nucleus and with redistribution of the nuclear transport factors Ran, importin-β and importin-α across the NE^34,36,46^. Notably, Bcl-2 expression increases NE permeability, and targeting it to the NE as a KASH domain fusion protein (also containing the transmembrane segment adjacent to the KASH domain) is sufficient to promote permeability^46^. In addition, an early event in apoptosis is NE dilation, which occurs in a caspase-independent manner^47^. Taken together, these findings show that changes in NE structure, increased permeabilization and the subsequent redistribution of proteins across the NE can be mediated in a caspase-independent process.

An additional caspase-independent effect of apoptosis is nuclear deformation induced by interaction between p53 and lamin A/C. p53 is known to be activated and to play a role in apoptosis induced by diverse stress stimuli^48^. Activated p53 can bind to and stabilize lamin A/C and induce NE deformations^49^.

To summarize, the apoptotic process targets the NE. This is mediated by caspase-dependent cleavage of NPCs, nuclear lamins, and other NE proteins, which leads to an increase in NE permeability and the subsequent entrance of apoptogenic cytosolic proteins into the nucleus. The consequences are NE breakdown and destruction of the nucleus. However, in addition to the caspase-dependent pathway, the apoptotic process can increase NE permeability in a caspase-independent manner.

## The NE as a mediator of the apoptotic process

### Bax-dependent and caspase-independent SIGRUNB and nuclear protein redistribution

**Fig. 3:**
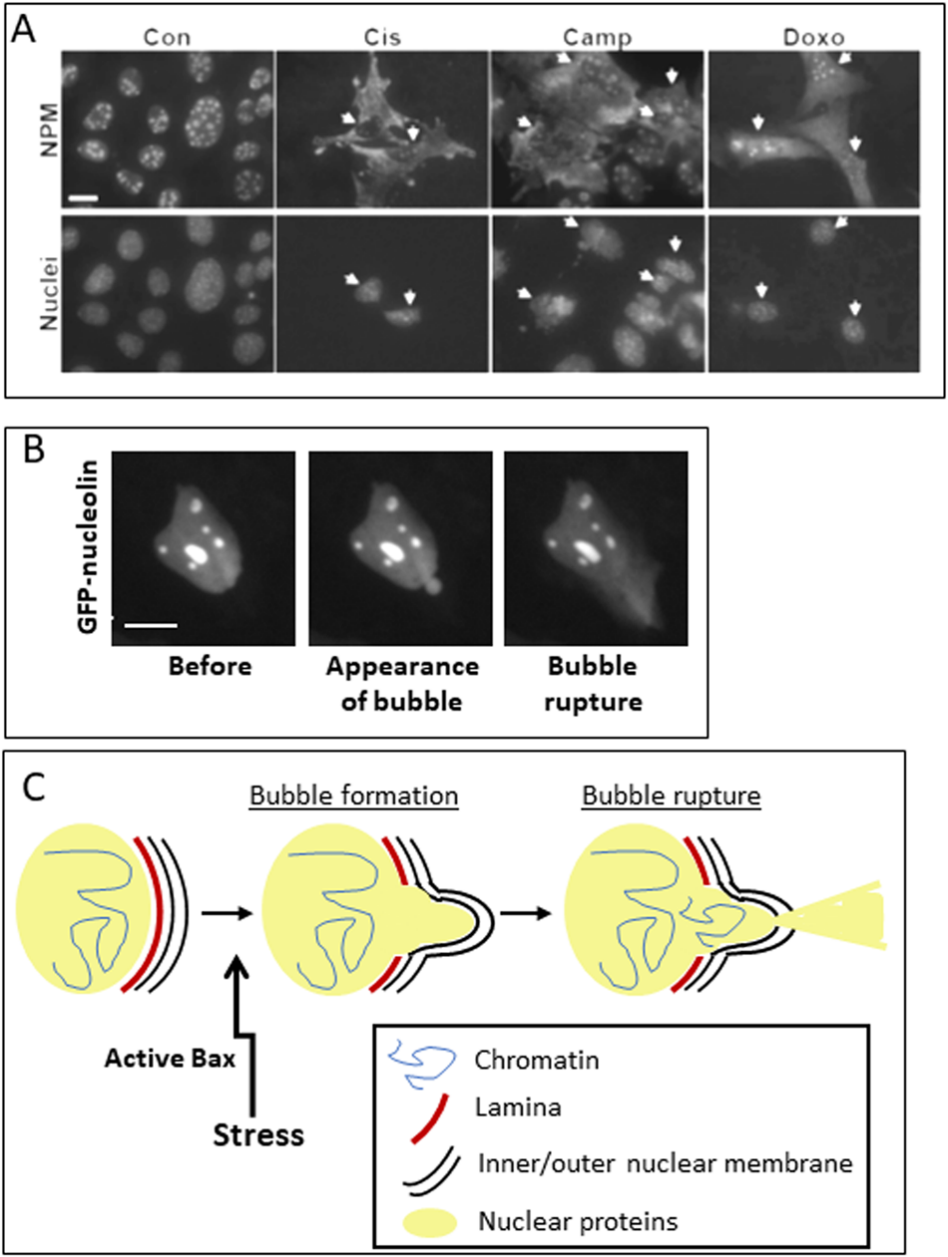
Stress-induced NPR and SIGRUNB. **a** Redistribution of the nuclear protein nucleophosmin (NPM) in response to apoptotic stimuli. Wild type MEFs were untreated (Con) or treated for 24 h with cisplatin (Cis), camptothecin (Camp), or doxorubicin (Doxo), followed by staining with anti-NPM antibodies and Hoechst 33258 (to label nuclei), and thereafter visualized by fluorescence microscopy. The images after each treatment (upper and lower panels) represent the same field visualized separately for NPM labeling and Hoechst-stained nuclei. Arrows indicate cells exhibiting the redistribution of NPM and their nuclei. Size bar = 20 µm. (Modified from Lindenboim et al.^50^). **b** SIGRUNB induced by cisplatin treatment. GFP-nucleolin-expressing MEFs were treated with cisplatin and imaged by fluorescence microscopy after 15–24 h. Arrow indicates a bubble and right panel shows subsequent rupture. Size bar = 10 µm (Modified from Lindenboim et al.^21^). **c** Schematic diagram of SIGRUNB. In response to apoptotic stimuli/stress, Bax induces NPR by a process involving local disturbances in NE proteins, including lamin A/C, which results in the generation and subsequent rupture of nuclear protein-containing bubbles. The bubbles do not contain DNA at early stages and are encapsulated by nuclear pore complex-depleted regions of the NE (bubble formation). At later times, the bubbles rupture and discharges nuclear proteins into the cytoplasm (bubble rupture).

Apoptosis-induced NE leakiness that leads to translocation of nuclear proteins from the nucleus to the cytosol can also occur by a Bax/Bak-dependent and caspase-independent manner^50,51^. Treatment of mouse embryonic fibroblasts (MEFs) with cisplatin, campothecin, or staurosporine leads to the appearance of the nuclear proteins nucleophosmin, nucleolin, and histone H1 in the cytosol (Fig. 3a). The nuclear protein redistribution (NPR) is regulated by Bax in a non-canonical manner. It does not occur in MEFs lacking Bax/Bak; however, re-expression of Bax restores the effect. Furthermore, NPR occurs before the appearance of late-onset apoptotic events, namely Bax/Bak activation, cytochrome *c* release, and caspase-3 activation. It also occurs independent of caspases and is unaffected by overexpression of the anti-apoptotic protein Bcl-x_L_. Moreover, localization of a Bax KASH domain fusion protein in the NE triggers NPR without inducing MOMP or apparent apoptotic cell death. Bax-induced NPR is associated with impairment in lamin A mobility, implying a connection between these two NE-associated events^50,51^.

This stress-induced NPR is likely mediated by NE rupture, as demonstrated by imaging of fluorescently labeled nuclear proteins in live cells. These fluorescent reporters are normally localized to the nucleus; however, in response to apoptotic triggers, they appear in bubbles protruding from the nuclear periphery. These bubbles then rupture and the fluorescent proteins are discharged into the cytoplasm (Fig. 3b). This process is transient, localized to foci at the nuclear periphery and repetitive; the bubbles are encapsulated by nuclear pore complex-depleted NE^21^. At early times following their generation, the bubbles do not contain DNA^21^. However, at later times, DNA enters the bubbles and in some cases spills out from them (unpublished observation Stein lab). Similar to NPR, this process, termed SIGRUNB (Fig. 3c), is Bax/Bak-dependent, preceded morphological changes of apoptosis, occurs independently of caspases and is not inhibited by Bcl-x_L_. Thus, NE rupture and subsequent NPR are mediated by a non-canonical action of Bax.

We currently do not know how Bax regulates SIGRUNB/NPR. One possible mechanism is that Bax mediates this effect from the NE. This assumption is supported by studies showing that Bax can be found in the membranes of the NE^52,53^. Furthermore, enrichment of Bax in the NE, mediated by fusion to a KASH domain, leads to SIGRUNB/NPR^21,51^. KASH domain-mediated targeting of Bcl-2 to the NE also promotes permeability^46^, implying a yet unknown similarity in the action of these two functionally different members of the Bcl-2 family. Both may lead to the direct perforation of the nuclear membranes, since in vitro studies indicate that both have channel prompting activity^54^. Alternatively, both may bind to a common target, such as a LINC complex protein, to promote NE rupture^55^.

GRUNB is not a unique feature of the apoptotic stress response, but can also occur in other systems. For example, it occurs in cells expressing HIV Vpr^56^, in *Drosophila* muscle cells during Wnt signaling^57^, during confined cell migration^58–61^ and in response to mechanical compression^62^. In some cells, GRUNB can also occur spontaneously, in the absence of an exogenous signal. Cells from patients with lamin A/C gene mutations or cells derived from tumors exhibit spontaneous and repeated NE ruptures accompanied by discharge of nuclear proteins into the cytosol^63–65^. In addition, the NE of MEFs lacking all lamins also undergoes frequent rupture^66^. GRUNB also appears in cultured cancer cells with reduced levels of lamin B1^64,67^ and in primary MEFs lacking this protein^68^.

GRUNB and SIGRUNB share common features. In both processes, the NE protrudes into buds that extend into nuclear bubbles. The bubbles contain nuclear proteins and DNA, although in SIGRUNB the DNA appears only at a later stage after the generation of the bubble. The bubbles of GRUNB and SIGRUNB rupture in a transient and repetitive manner and NE surrounding them is devoid of NPCs^21,56,58,63,64^ as well as other NE proteins. For example, emerin is absent from bubbles induced by apoptotic stress (SIGRUNB) and LINC components are absent from bubbles formed in some interphase cells^55,64^. Another common feature of GRUNB and SIGRUNB is disruptions of the nuclear lamina. Apoptotic stress impairs lamin A/C mobility in a caspase-independent manner^21^ and reduces it at the bubble site (Fig. 3c)^21^. HIV-1 Vpr impairs lamin C structure at the site of a bubble^56^, and constricted migration causes a near complete loss of B-type lamins as well as dilated webs of lamin A/C at the bubble site^61^. These alterations in lamins are consistent with the observations that expression of pathogenic lamin A variants or their downregulation^63^, a decrease in lamin B1 expression^55^ or depletion of all lamins^64^ promotes or increases NE rupture. Rupture then leads to redistribution of nuclear proteins from the nucleus to the cytosol^56,61,63,64^, and from the cytosol to the nucleus^58,63,64^, although the latter has not yet been examined in SIGRUNB. GRUNB is repetitive most likely because the NE can be repaired after rupture. This has been shown in the case of constricted migration-induced GRUNB, where the ESCRT-III repair machinery is recruited to NE rupture sites and helps restore integrity^69^. The role of ESCRT-III in SIGRUNB has not been studied, but the repetitive nature of bubble formation and rupture implies that such a repair process may also occur.

Although SIGRUNB and GRUNB share many features, there are few subtle differences between them. In apoptotic stress-induced bubbles, lamin A/C is excluded from the site of nuclear bubble protrusion and early stage bubbles lack DNA^21^. In contrast, constricted migration-induced nuclear bubbles contain lamin A/C, although in variable amounts^58,61^, as well as DNA and lamin B2, but not lamin B1^58^. Bubbles induced by HIV Vpr^56^ and in interphase cancer cells also contain DNA^64^, and the rupture of the latter occurs at sites with low amounts or absence of lamin B1^63^. Whether these differences are due to differences in the cell types and GRUNB’s inducers still needs to be addressed.

Notably, transient NE rupture may not occur only via GRUNB. Accordingly, fibroblasts lacking nuclear lamins do not exhibit nuclear bubble or protrusions but display frequent and prolonged nuclear membrane ruptures, associated with DNA damage and occasionally by cell death^66^. Whether this process may occur also in response to apoptotic trigger still need to be examined.

#### Consequence of SIGRUNB/NPR

The direct role of SIGRUNB/NPR in cell death has not been studied. However, it is feasible that this process can contribute to the death process. Although it may not directly cause cell death, the repetitive nature of SIGRUNB/NPR may irreversibly damage the NE and the nucleoplasm. This notion is supported by the findings that repetitive NE ruptures induced by lack of a nuclear lamina^66^ or during constricted migration can cause cell death^59^. Furthermore, constricted migration-induced NE rupture is associated with translocation of DNA repair factors from the nucleus to the cytosol as well as with DNA damage^58,61^. In this setting, damaged DNA cannot be properly repaired due to the mislocalization of repair factors from the nuclei. The fate of DNA repair factors has not been examined in SIGRUNB. However, in view of its similarity to migration-induced GRUNB, it is feasible that mislocalization of DNA repair factors may also occur during SIGRUNB, in turn enhancing apoptosis-mediated DNA damage. In addition, caspase-independent, Bax-dependent NPR redistributes nuclear proteins^50^ known to promote cell death, such as nucleophosmin^70–73^. Thus, SIGRUNB may amplify the apoptotic process, ensuring that cell death will be executed when initiated by activated Bax, but cannot occur because caspase activity is blocked. This may occur with the expression of high amounts of inhibitors of apoptosis proteins.

Lastly, GRUNB/SIGRUNB might also cause pyroptosis^74^, a regulated form of necrotic cell death. Accordingly, in response to GRUNB/SIGRUNB, DNA can leak from the nucleus to the cytosol. It is also known that cytosolic double-stranded DNA (dsDNA) binds to and directly activates the inflammasome-initiating sensors, AIM2, and this in turn induces the formation of the inflammasome, a caspase-1 activation platform^75^. Active caspase-1 can promote pyroptosis by cleaving and activating the pore-forming effector protein gasdermin D^76^, and inflammation by activating and promoting the release of the pro-inflammatory cytokines IL-1β and IL-18^75^. With respect to inflammation, in analogy to the inflammatory effect of DNA released from the mitochondria to the cytosol in response to Bax/Bak-dependent MOMP, nuclear dsDNA released into the cytosol may promote inflammation by activating the cGAS-STING-dependent type I interferon inflammatory response^77,78^.

#### The role of the LINC complex in apoptosis-induced NE leakiness/rupture

Cytoskeletal forces are transmitted to the nucleus via the LINC complexes^20,79–81^. Non-apoptotic nuclear rupture may be caused by contractile forces imposed on the NE by actomyosin fibers via the LINC complex^55^. Consistent with this hypothesis, depleting the LINC complex protein SUN1 reduces the frequency of chromatin herniation (an indication of interphase NE rupture^55^) in cells with pathological alterations in lamin A/C^82^. Furthermore, NE rupture in cancer cells with reduced lamin B1 expression depends on the assembly of contractile actin fibers and the LINC complex^55^. Consistent with a potential role of the LINC complex in NE defects, knockdown of nesprin-2 causes convolutions of the NE, loss of NE integrity and vesiculation of the ONM^83^. The cytoskeleton is subjected to reorganization during apoptosis^84^. This in turn may impose force transmitted by the LINC complex on the nucleus, which in combination with caspase-dependent or independent weakening of the nuclear lamina may cause GRUNB. This assumption is supported by the finding that in apoptotic cells, caspase-mediated cleavage of Rho‐associated kinase 1 leads to its activation, which then promotes contraction of the actin‐myosin cytoskeleton and tearing of the NE^85^. This effect may be mediated by coupling actin-myosin filaments to the LINC complex proteins nesprin-1 and nesprin-2^85^. In addition, force exerted through focal adhesions causes nuclear deformations in a process mediated by stress fibers and LINC complexes. This in turn stretches nuclear pores, directly driving the nuclear translocation of the transcription factor YAP/TAZ by decreasing the mechanical restriction of NPCs to molecular transport^86^. A similar effect may promote the early caspase-independent increase in NE leakiness observed during apoptosis^46,85^. Additional evidence for the role of the LINC complex in NE leakiness/ruptures comes from the observation that microtubule destruction plays an important role in NE breakdown induced by C53/LZAP-C3^44^. Since the LINC complex proteins nesprin-1 and nesprin-2 can bind microtubules via their associations with the motor proteins kinesin and dynein^87,88^, it is possible that the effect of C53/LZAP-C3 on the NE is mediated via alterations in the association between microtubules and the LINC complex.

To summarize, the apoptotic process via non-canonical action of Bax can cause local and transient alteration in the NE, which in turn leads to generation and subsequent rupture of nuclear bubbles. This process, which might be mediated via the LINC complex, causes redistribution of nuclear proteins to the cytosol, which in turn may amplify the apoptotic process by several pathways.

### Regulatory role of the NE on the apoptotic machinery

#### Pro-apoptotic and anti-apoptotic activities of NE proteins

Besides enabling translocation of apoptogenic factors into and from the nucleus during SIGRUNB/GRUNB, the NE may also regulate cell death by other means. Emerin may act as an anti-survival protein by interfering with Notch signaling. Emerin can interact with the Notch intracellular domain (NICD) at the NE and thereby prevent it from acting on target genes. This in turn impedes Notch-mediated survival signaling. In addition, knockdown of the emerin-binding proteins lamin A/C and barrier-to-autointegration factor releases emerin to the ER and enhances Notch signaling^89^. Thus, in cells that are engaged in Notch signaling, apoptotic stress may also activate an anti-apoptotic pathway by caspase-mediated proteolysis of lamin A/C, which will deplete emerin from the NE and free NICD from its inhibitory effect. This assumption is supported by a study that showed that ER stress, a known apoptotic trigger^90^, clears emerin from the INM and ER, although by a different mechanism^91^. Pro-apoptotic activity can also be promoted by SUN2, an INM component of the LINC complex. Overexpression of SUN2 increases apoptosis, whereas knockdown of SUN2 reduces it in naïve and cisplatin-treated lung cancer cells^92^. The pro-apoptotic effect of SUN2 may not be mediated via the LINC complex, since the ONM components of the LINC complex, nesprin-1 and nesprin-2, may have anti-apoptotic activities. Knockdown of nesprin-1^93^ or nesprin-2^94^ can cause cell death. The mechanism whereby the different components of the LINC complex interact with the apoptotic machinery still need to be elucidated.

#### The NE as a platform for recruitment and assembly of apoptosis-promoting complexes

The NE may serve as a platform to recruit apoptosis-promoting complexes. In healthy cells of the nematode *Caenorhabditis elegans*, the pro-apoptotic protein, cell death gene (CED)-4, is neutralized by binding to the anti-apoptotic protein CED-9 in the mitochondria. In cells doomed to die, expression of the BH3-only protein EGL-1 is induced and then binds to CED-9 in the mitochondria. This in turn releases CED-4 from its interaction with CED-9. Once released, CED-4 translocates from the mitochondria to the NE. This translocation promotes the cell death process in a CED-3-independent manner^95^. Notably, the SUN-domain protein matefin/SUN-1 is required for CED-4 localization at the NE^96^. Moreover, CED-3 localizes to the perinuclear region in *C. elegans* germ cells, providing an explanation for the translocation of CED-4 to the NE during apoptosis. The perinuclear localization of CED-3 and CED-4, and CED-3 zymogen autoactivation is regulated by the *C elegans* nuclear-pore protein NPP-14^97^.

Assembly of apoptotic complexes in the NE may also occur in mammalian cells. Treatment of human cervical carcinoma C4-I cells with etoposide generates an NE-associated apoptotic promoting complex^98^. In response to an apoptotic trigger, PKCζ is translocated to NE followed by recruitment of Bcl10 to NE-associated PKCζ. Bcl10 then recruits 3-phosphoinositide-dependent protein kinase-1 (PDK1) to the PKCζ•Bcl10 complex. PDK1 phosphorylates and activates PKCζ, which in turn phosphorylates Bcl10. Phosphorylated Bcl10 nucleates a second complex (or expands the first one) composed of PKCζ and active caspase-3. PKCζ promotes caspase-3 phosphorylation, which enhances caspase-3 proteolytic activity at NE^98^.

## Conclusions

**Fig. 4:**
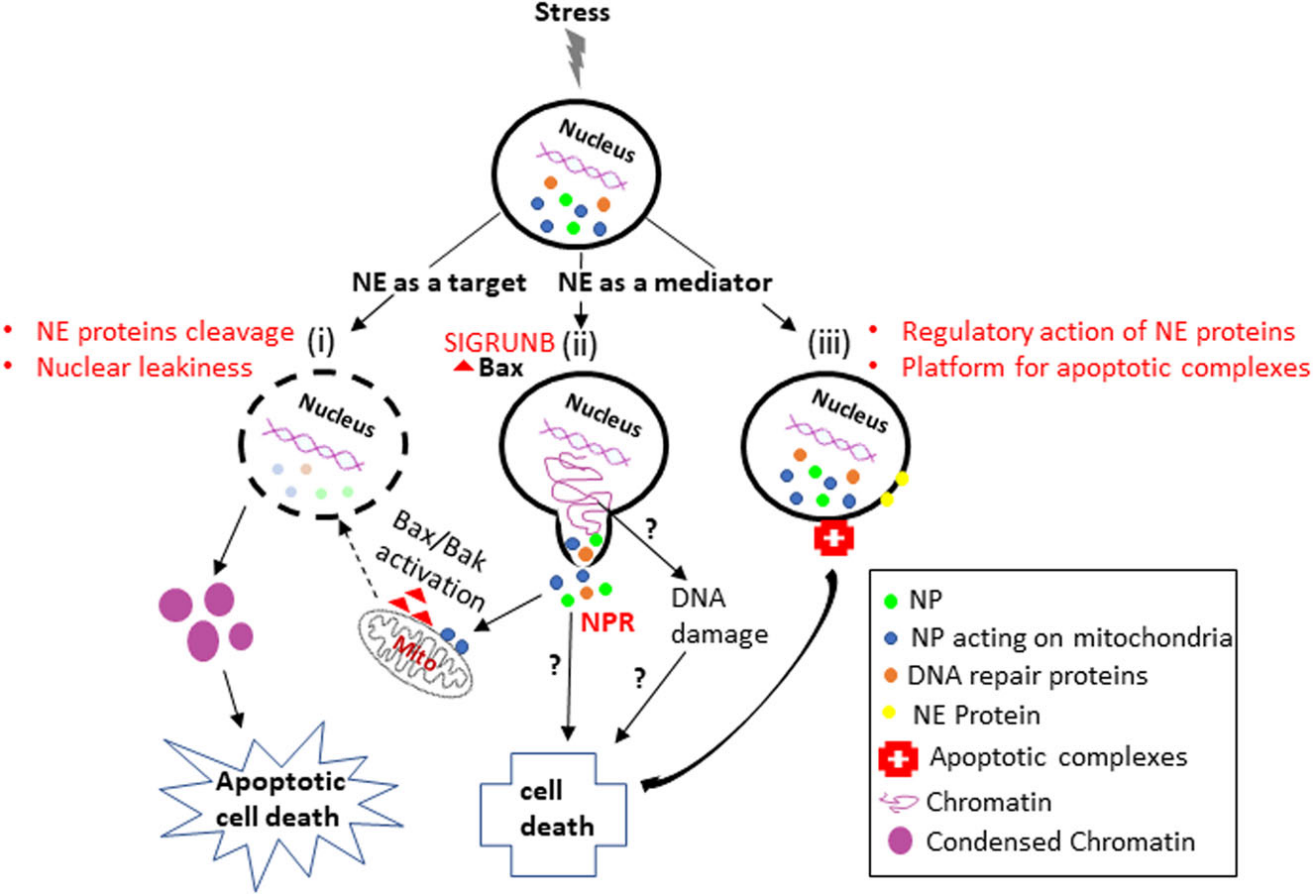
The NE is both a target and a mediator of the apoptotic process. Apoptotic stress converges on the NE where it promotes two major effects. (i) Caspase-dependent cleavage of NPC proteins and lamins. This in turn causes NE leakiness and entrance of caspases and nucleases from the cytosol to the nucleus, leading to NE breakdown and chromatin condensation and fragmentation. This nuclear destruction, along with additional non-nuclear events, culminate in apoptotic cell death. (ii–iii) Mediation of the death process. This effect includes (ii) Bax (red triangle)-dependent and caspase-independent transient SIGRUNB, which leads to NPR from the nucleus to the cytosol and perhaps also nuclear DNA damage. Some nuclear proteins such as nucleophosmin and histone 1.2 can translocate to the mitochondria and activate Bax/Bak, leading to MOMP. DNA repair factors are also released during SIGRUBN, depriving the nucleus of the ability to repair damage, further contributing to cell death. Other nuclear proteins released to the cytosol may also contribute to cell death by yet unknown mechanisms. (iii) Regulatory role of the NE on the apoptotic machinery. NE proteins can have pro- and anti-apoptotic effects and thus may regulate the apoptotic process. Furthermore, the NE can serve as a platform for the recruitment and assembly of apoptosis-promoting complexes. Uncertainties are indicated by a “?”.

Intracellular membranes play a major role in apoptosis. Of these, the mitochondrial outer membrane is the most studied, since its perforation plays an essential role in the Bcl-2 family-regulated intrinsic apoptotic pathway (for a recent review see Kalkavan and Green^99^). However, the NE is also emerging as an important module of the death process, acting both as a target for destruction as well as a mediator (Fig. 4). Targeting the NE leads to a highly efficient and rapid manifestation of nuclear apoptosis. The caspase-dependent destruction of the NPCs and the nuclear lamina perturb the NE barrier and its association with the chromatin. This in turn enables translocation of apoptotic factors to the nucleus, which then mediate its destruction. This process may occur at the late apoptotic stage following caspase activation and is associated with NE breakdown.

However, the NE is not only a target but can participate in mediating the apoptotic process. One example is caspase-independent, Bax-dependent SIGRUNB, which occurs at an early stage of the cell death process. SIGRUNB and the associated NPR leads to translocation of several nuclear proteins from the nucleus to the cytosol, some of which can promote apoptosis via the mitochondrial pathway^50,70–73,100,101^. SIGRUNB might also deplete the nucleus of DNA repair factors, as shown to occur during constricted cell migration^61^. This in turn may also contribute to cell death by accelerating apoptosis-induced DNA damage. Repetitive GRUNB can also cause cell death^59,66^.

We hypothesize that SIGRUNB acts as an amplifier of the apoptotic process, which may be particularly relevant in cells in which caspase activity is constantly inhibited. The NE may also regulate apoptosis by the action of some of its proteins that can exert anti- or pro-apoptotic effects as well as by serving as a docking site for recruiting apoptosis-promoting complexes^95,98^. Via these mechanisms, the NE may therefore act as an amplifier of the apoptotic process. Additional studies aimed at assessing the mechanism whereby Bax mediates SIGRUNB and the effect of SIGRUNB-mediated NPR on the apoptotic pathways should increase our understanding of this new non-canonical action of Bax and novel cell death pathways.
